# Supplementary material for: Targeting the Class A Carbapenemase GES-5 via Virtual Screening
Source: Biomolecules. 2020 Feb 14;10(2):304. doi: 10.3390/biom10020304 (PMC7072645; doi:10.3390/biom10020304)
Supplement: Supplementary file 1 [file biomolecules-10-00304-s001.pdf]

biomolecules

MDPI

Supplementary material

Targeting the Class A Carbapenemase GES-5 via Virtual Screening

Raphael Klein [1], Laura Cendron [2], Martina Montanari [3], Pierangelo Bellio [4], Giuseppe Celenza [4], Lorenzo Maso [2], Donatella Tondi [3,*] and Ruth Brenk [5,*]

[1] Institute of Pharmacy and Biochemistry, Johannes Gutenberg University, Mainz, Germany; raphael.klein@outlook.de

[2] Department of Biology, University of Padua, Viale G. Colombo 3, 35121, Padua, Italy; laura.cendron@unipd.it (L.C.); lorenzo.maso@studenti.unipd.it (L.M.)

[3] Department of Life Sciences, University of Modena and Reggio Emilia, Via Campi 103, 41125, Modena, Italy; 186334@studenti.unimore.it (M. M.)

[4] Department of Biotechnological and Applied Clinical Sciences, University of L'Aquila, via Vetoio 1, 67100 L'Aquila, Italy; pierangelo.bellio@univaq.it (P.B.); giuseppe.celenza@univaq.it (G.C.)

[5] Department of Biomedicine, University of Bergen, Jonas Lies Vei 91, 5020 Bergen, Norway

* Correspondence: tondi.donatella@unimore.it (D.T.); ruth.brenk@uib.no (R.B.); Tel.: +39-05-9205-8577 (D.T.); +47-5558-6070 (R.B.)

Received: 14 January 2020; Accepted: 11 February 2020; Published: 13 February 2020

Table S 1: Data collection and refinement statistics.

	**GES-5**
**PDB code**	6TS9
**Wavelength**	0.97624
**Resolution range**	55.33 - 1.55 (1.58 - 1.55)
**Space group**	P 21 21 21
**Unit cell**	76.3 80.36 87.85 90 90 90
**Unique reflections**	78894 (7770)
**Multiplicity**	6.0 (5.7)
**Completeness (%)**	99.94 (99.96)
**Mean I/sigma(I)**	18.96 (2.69)
**Wilson B-factor**	20.08
**R-merge**	0.04645 (0.5643)
**CC1/2**	1 (0.876)
**Reflections used in refinement**	78892 (7770)
**Reflections used for R-free**	3915 (394)
**R-work**	0.1795 (0.2364)
**R-free**	0.2037 (0.2740)
**CC(work)**	0.958 (0.835)
**CC(free)**	0.926 (0.808)
**Number of non-hydrogen atoms**	4829
**macromolecules**	4271
**ligands**	16
**solvent**	542
**Protein residues**	537
**RMS(bonds)**	0.014
**RMS(angles)**	1.94
**Ramachandran favored (%)**	97.94
**Ramachandran allowed (%)**	1.88
**Ramachandran outliers (%)**	0.19
**Rotamer outliers (%)**	4.09
**Clashscore**	7.27
**Average B-factor**	24.44
**macromolecules**	22.99
**ligands**	34.49
**solvent**	35.57

Data for the highest-resolution shell are shown in parentheses.

Table S 2: Compound codes, chemical structures and inhibition data for purchased compounds.

Code	Structure	MolPort ID	MW	% inhibition at [1mM] *vs* GES-5	% inhibition at [1mM] *vs* KPC-2
**1**	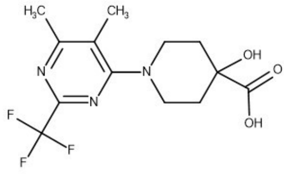	MolPort-020-216-167	319.284	19%	*n.d.*
**2**	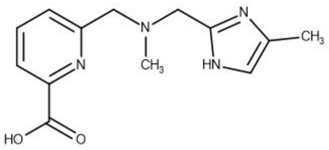	MolPort-030-047-117	260.297	21%	26%
**3**	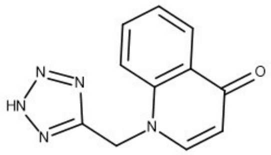	MolPort-007-988-798	227.227	18%	*n.d.*
**4**	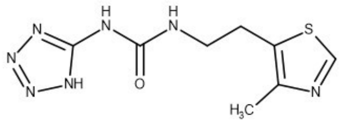	MolPort-005-142-722	253.28	23%	19%
**5**	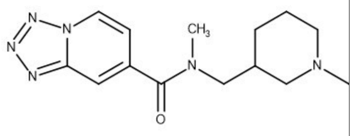	MolPort-020-210-470	302.382	27%	*n.d.*
**6**	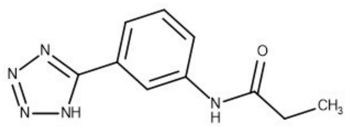	MolPort-002-129-311	217.232	33%	30%
**7**	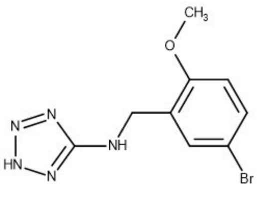	MolPort-000-864-739	284.117	21%	19%

**8**	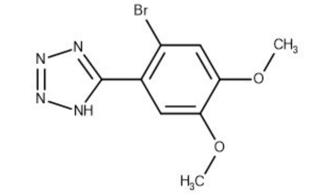	MolPort-002-104-350	285.101	IC$_{50}$ 1.74	IC$_{50}$ 2.31
**9**	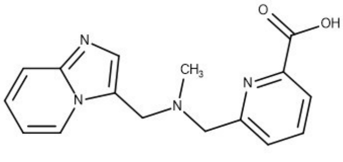	MolPort-035-370-005	296.33	16%	*n.d.*
**10**	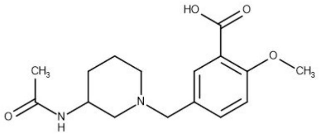	MolPort-035-383-766	306.362	19%	19%
**11**	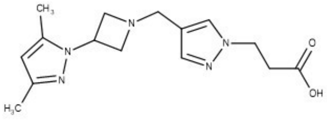	MolPort-030-060-088	303.366	*Not tested because of solubility issues*	
**12**	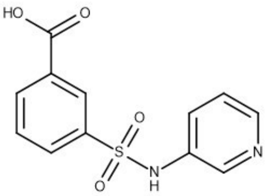	MolPort-002-307-735	278.28	IC$_{50}$ 1.98	IC$_{50}$ 2.71
**13**	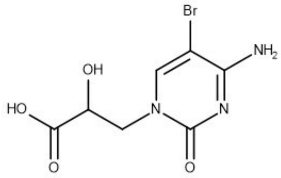	MolPort-003-884-547	278.062	IC$_{50}$ 1.58	IC$_{50}$ 1.86
**14**	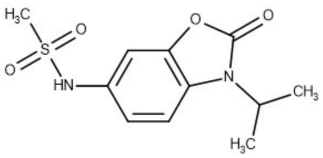	MolPort-007-617-735	270.3	10%	*n.d.*

**15**	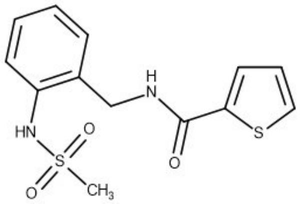	MolPort-009-318-942	310.39	16%	*n.d.*
**16**	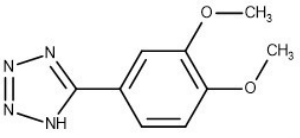	MolPort-001-005-590	206.205	*n.d.*	*n.d.*
**17**	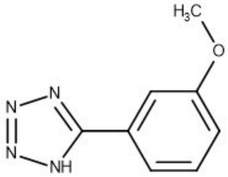	MolPort-002-467-448	176.179	28%	17%
**18**	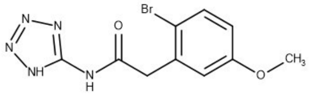	MolPort-027-913-957	312.127	21%	18%
**19**	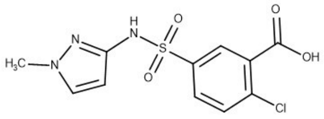	MolPort-009-422-837	315.73	34%	20%
**20**	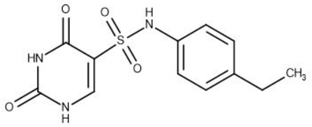	MolPort-003-130-164	295.31	26%	24%
**21**	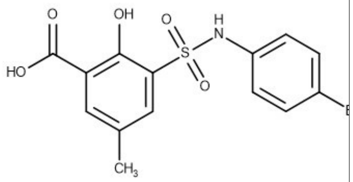	MolPort-009-350-762	386.22	32%	24%
**22**	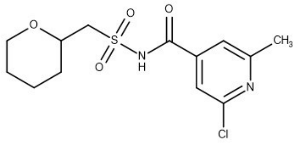	MolPort-035-847-366	332.8	18%	21%

**23**	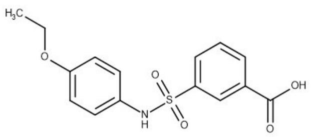	MolPort-002-305-656	321.35	29%	44%
**24**	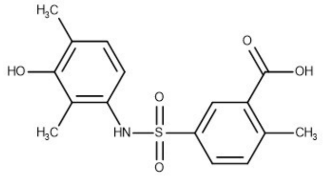	MolPort-009-556-594	335.37	32%	31%
**25**	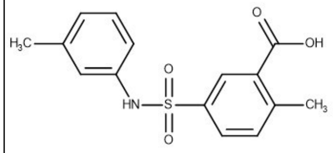	MolPort-002-462-443	305.35	23%	28%
**26**	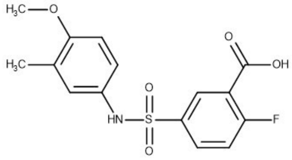	MolPort-020-063-552	339.34	30%	30%
**27**	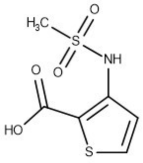	MolPort-009-460-556	221.25	19%	29%
**28**	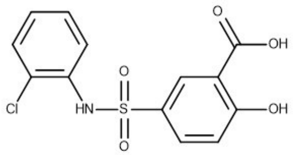	MolPort-002-363-286	327.74	29%	40%
**29**	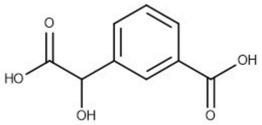	MolPort-021-943-905	196.158	19%	22%

**30**	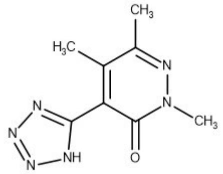	MolPort-005-311-448	206.209	*Not tested because of solubility issues*	
**31**	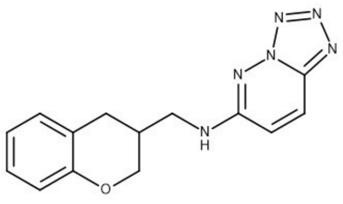	MolPort-009-435-655	282.307	*Not tested because of solubility issues*	
**32**	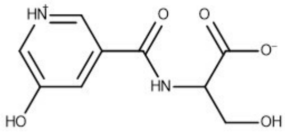	MolPort-000-768-609	226.188	21%	14%
**33**	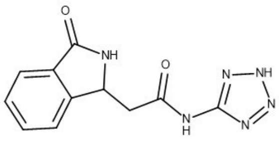	MolPort-027-846-104	258.241	20%	20%
**34**	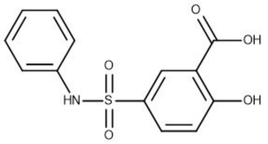	MolPort-000-644-251	293.29	*Not tested because of solubility issues*	
**35**	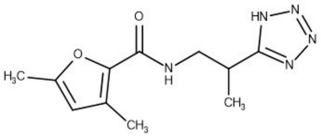	MolPort-030-031-849	249.274	24%	27%
**36**	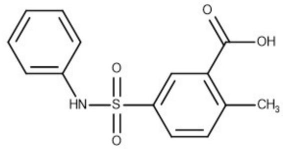	MolPort-005-178-145	291.32	21%	*n.d.*
**37**	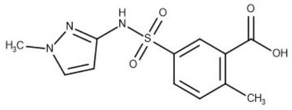	MolPort-005-705-646	295.31	23%	*n.d.*

**38**	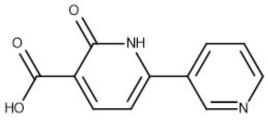	MolPort-006-756-424	216.196	*Not tested because of solubility issues*	
**39**	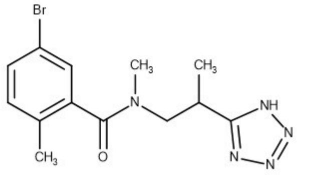	MolPort-035-750-522	338.209	21%	*n.d.*
**40**	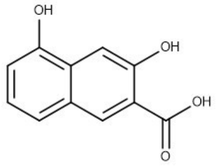	MolPort-001-790-951	204.181	23%	*n.d.*
**41**	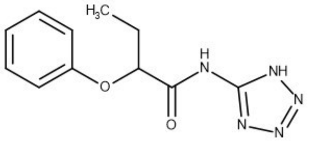	MolPort-001-544-523	247.258	33%	24%
**42**	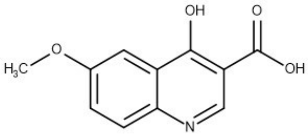	MolPort-000-004-269	219.196	*Not tested because of solubility issues*	
**43**	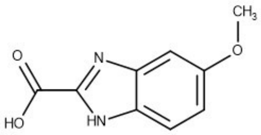	MolPort-019-878-939	192.174	IC_{50} 1.29	IC_{50} 1.88
**44**	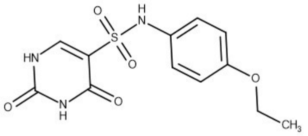	MolPort-003-130-166	311.31	23%	*n.d.*

n.d: not determined
